# PCSK9 and Lipid Metabolism: Genetic Variants, Current Therapies, and Cardiovascular Outcomes

**DOI:** 10.1007/s10557-024-07599-5

**Published:** 2024-06-22

**Authors:** Daniela Grejtakova, Iveta Boronova, Jarmila Bernasovska, Stefano Bellosta

**Affiliations:** 1 Laboratory of Molecular Genetics, Department of Biology, Faculty of Humanities and Natural Sciences, University of Presov, 17 November 1, Presov, 08001 Slovakia; 2 Department of Pharmacological and Biomolecular Sciences “Rodolfo Paoletti”, Università Degli Studi di Milano, Via Balzaretti 9, 20133 Milan, Italy

**Keywords:** *PCSK9*, Genetic variants, Lipoproteins, Cardiovascular events, PCSK9 inhibitors, Meta-analysis

## Abstract

Proprotein convertase subtilisin/kexin type 9 (*PCSK9*) plays a crucial role in the modulation of lipid metabolism as a critical negative regulator of hepatic low-density lipoprotein receptor (LDLR) levels and circulating low-density lipoprotein (LDL) clearance. Numerous gain-of-function (GOF) mutations in *PCSK9* have been identified as causing familial hypercholesterolemia (FH) by reducing LDLR levels, and loss-of-function (LOF) mutations associated with a hypercholesterolemia phenotype protective against atherosclerosis. *PCSK9* represents an example of successful translational research resulting in the identification of *PCSK9* as a major drug target for a lipid-lowering therapy. To explore the genetic constitution of *PCSK9* and its biologic role, in this review, we summarize the current evidence of clinically significant *PCSK9* genetic variants involved in lipid metabolism as well as emphasize the importance of PCSK9 inhibition for the improvement of cardiovascular outcomes by conducting a meta-analysis of the available data on the incidence of cardiovascular disease events.

**Supplementary Information:**

The online version contains supplementary material available at 10.1007/s10557-024-07599-5.

## Introduction

Cardiovascular disease (CVD) due to atherosclerosis is the foremost cause of premature mortality worldwide. It is generally accepted that elevated blood lipids play an important role in the development of atherosclerotic plaques and cardiovascular disease etiopathogenesis. Over the last two decades, the field of lipid study has paid attention to the study of proprotein convertase subtilisin/kexin type 9 (*PCSK9*)*. PCSK9* is one of the three most important genes involved in familial hypercholesterolemia, other than LDLR or apolipoprotein B (APOB). While *PCSK9* gain-of-function (GOF) mutations associated with elevated blood lipids, carriers of loss of-function (LOF) mutations benefited from low-density lipoprotein cholesterol (LDL-C) reduction by up to 28%, accompanied by an 88% reduced risk of coronary artery disease [1]. Hepato-specific reduction LDL-C levels remains the first strategy in managing patients with familial hyperlipidemia and those with clinical atherosclerotic cardiovascular disease not reaching lipid-reducing goals [2]. To mimic *PCSK9* natural inhibition, in 2015 PCSK9 monoclonal antibodies, the first strategy to inhibit PCSK9 therapeutically successfully approved in addition to statin therapy. More recently, silencing RNA (siRNA) reduced LDL-C by 45–60% [3]. Here, we systematically reviewed the available scientific evidence of genetic variation on *PCSK9* and assessed the efficacy of PCSK9 inhibitors in cardiovascular outcomes, including cardiovascular death, myocardial infarction, and stroke.

### PCSK9: Biological Function

PCSK9, the ninth member of the proprotein convertase family, is a serine protease that caught the attention of the scientific community in 2003 when the discovery of the first natural mutants of *PCSK9* revealed the implication of an as-yet-unknown actor in cholesterol homeostasis [4, 5]. *PCSK9* is mainly expressed on hepatocytes surface and has been shown to act both intracellularly playing a role as a chaperone in the degradation of the LDL receptor (LDLR), as well as a secreted factor promoting LDLR internalization from the hepatocellular surface [6]. PCSK9 regulates the degradation of the LDLR in response to cholesterol levels within the cell [7].

**Fig. 1:**
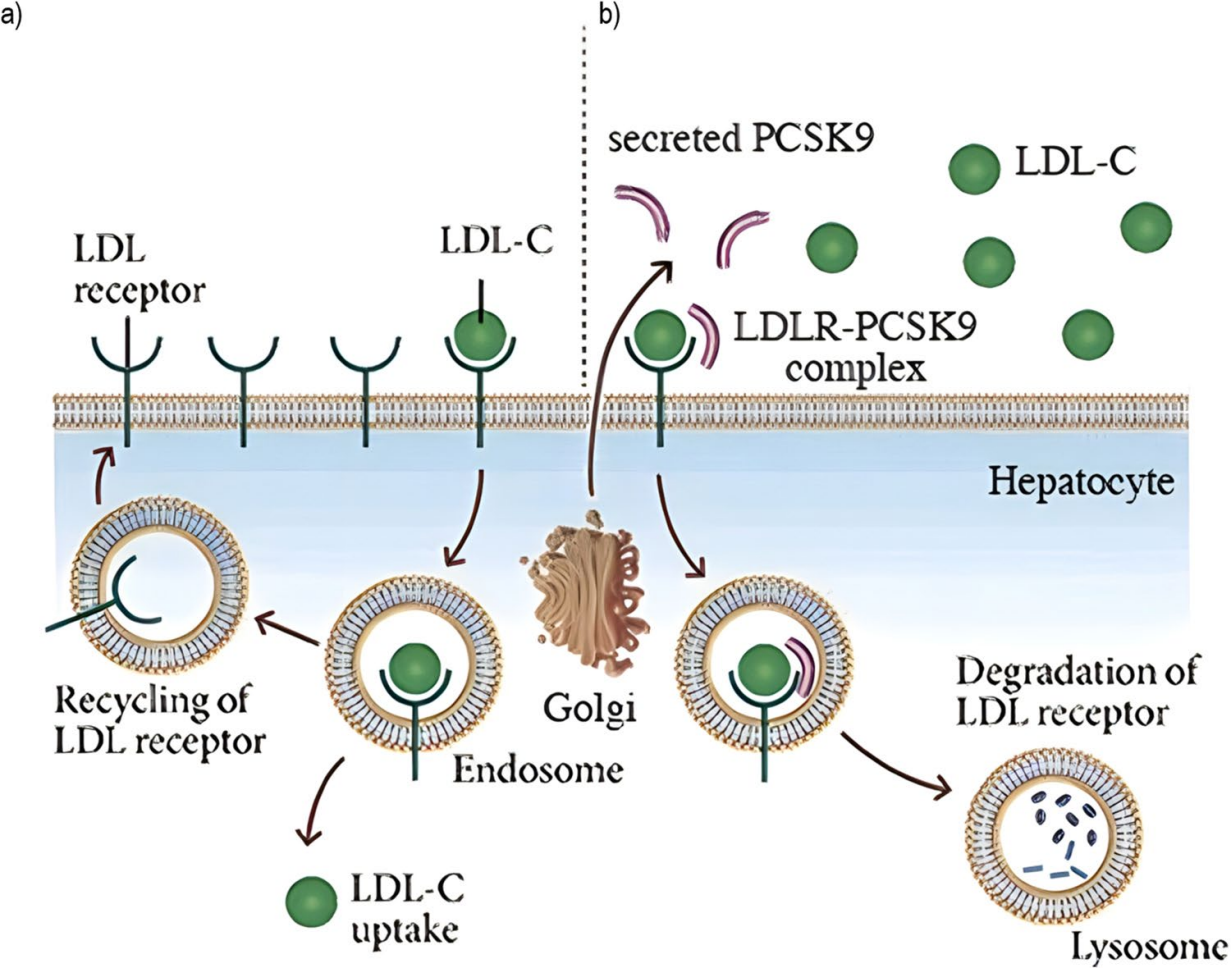
**a** LDL-C uptake and recycling of LDLR. **b** PCSK9-mediated degradation of LDLR. The protein PCSK9 regulates the number of LDL receptors (LDL-Rs) on a cell’s surface. When PCSK9 binds to these receptors, the receptors do not get recycled but are broken down in cellular compartments called lysosomes instead. Adapted with permission from Publisher [10]

PCSK9 protein structure is characterized by a signal sequence, a prodomain, and a catalytic domain, followed by a C-terminal region. PCSK9 is synthesized as an inactive 75 kDa proenzyme that undergoes autocatalytic cleavage in the endoplasmic reticulum (ER) which produces an approximately 60-kDa catalytic fragment. Autocatalytic cleavage of the zymogen in the ER is essential for its transport from this compartment and for its secretion. PCSK9 favors LDLR degradation independently of its catalytic activity by involving mainly extracellular and possibly intracellular pathways [8]. PCSK9 catalytic domain (aa153–421) strongly interacts with the EGF-A domain (aa314–355) of the LDLR (extracellular domain, ECD). This prevents the LDLR from forming a closed conformation, making the receptor susceptible to enzymatic degradation, rather than being recycled to the cell surface (Fig. 1) [9].

### PCSK9 Genetic Variants

**Fig. 2:**
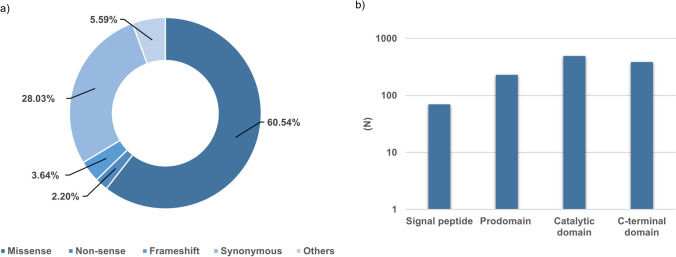
Number of genetic variants in *PCSK9* (transcript ID ENST00000302118.5) based on their functional effect: **a** distribution of mutations per domain; **b** types of mutation [11]

The human *PCSK9* gene is located on chromosome 1p32, and consist of 12 exons, which encode 692 amino acid protein (NP_777596.2). *PCSK9* gene is highly polymorphic with the distribution of genetic variants in all its domains affecting PCSK9 synthesis, secretion, and activity [11–13] (Fig. 2). In fact, gain-of-function (GOF) mutations occurred less frequent than loss-of-function (LOF) mutations.

Major findings confirmed the significant impact of *PCSK9* genetic variants on plasma LDL-C concentrations by affecting the LDLR pathway [4, 14, 15]. Beyond LDLR, PCSK9 affects also other lipoproteins, lipoprotein(a) [Lp(a)], and HDL-cholesterol levels are associated with circulating PCSK9 levels [16–19]. *PCSK9* may also play a role in the postprandial phase by modulating triglyceride-rich lipoprotein (TGRL) metabolism [20] (Supplementary file 1).

#### Gain-of-Function Mutations

Carriers of GOF *PCSK9* mutations over-express PCSK9, and therefore have very high plasma levels of LDL-C due to increased LDLR degradation. Several GOF missense mutations have been associated with hypercholesterolemia and premature atherosclerosis with overt CVD, such as myocardial infarction (MI) and stroke (S127R; F216L, D374Y, N157K; E32K; E228K; F216L, R357H, R215H, R496W, N35Y) [4, 5, 14, 15, 21–27].

Variant p.Ser127Arg interferes with autocatalytic cleavage, which is crucial for secretion from the cell. The p.Arg218Ser, p.Phe216Leu, and p.Asp374Tyr mutations result in total (p.Arg218Ser) or partial loss of the furin/PC5/6A processing of PCSK9, which increases the stability of PCSK9 [28, 29]. *PCSK9* S127R GOF mutation carriers demonstrated affected TG and LDL metabolism with a threefold increased production rate of apoB-100, compared with controls or LDLR-mutated patients, which led to direct overproduction of VLDL (threefold), intermediate-density lipoprotein (IDL, threefold), and LDL (fivefold) [30].

Elevated fasting plasma TG levels and increased cardiovascular risk were confirmed in carriers of another GOF *PCSK9* mutation E670G (− 23968A > G, rs505151) located in exon 12 [12, 31]. *PCSK9* E670G polymorphism is mainly associated with some serum lipid parameters in the Han population. The G allele carriers had higher serum HDL-C and apoAI levels in males, lower serum apoB levels, and higher apoAI/apoB ratio in females than the non-carriers of the G allele [32]. Interestingly, when *PCSK9* mutations were investigated in a Canadian Caucasian population, carriers of the E670G variant showed a non-significant difference in serum PCSK9 but a significantly lower TG concentration (27.7%; *p* = 0.039), not observed even for LOF mutations [33]. In a Japanese population, *PCSK9* GOF E32K was associated with over 30% increased plasma levels of PCSK9, whereas these results were confirmed also by media from transiently transfected HepG2 cells, compared to controls. In patients, homozygotes for the E32K mutation was a twofold higher LDL-C levels always present with a mutation in LDLR, and in the heterozygous carriers, almost half the effect was observed. This could suggest a *PCSK9* E32K effect on LDL-C levels via increased mass and function of *PCSK9* and could exacerbate the clinical phenotypes of patients carrying LDLR mutations [23]. Other *PCSK9* GOF mutants (F216L, R357H, and R215H) were described in different FH French families with tendon xanthomas, CHD, premature MI, and stroke [4, 5, 22]. GOF mutation in catalytic domain R215 was described to segregate with hypercholesterolemia in the Norwich family [24]. D374H was detected in one Portuguese proband and R496W in an Italian proband, together with E228K in LDLR [25, 26]. Another study demonstrated that variant p.Leu108Arg exhibited a ∼twofold-enhanced degrading activity towards the LDLR, resulting in a GOF, while variant p.Asp35Tyr created a novel Tyr-sulfation site, which may enhance the intracellular activity of *PCSK9* [14]. GOF mechanism could represent a higher activity of the LDLR-degrading function of *PCSK9*. However, a more recent hypothesis suggests that post-translational modifications within residues 31–60 may affect the inhibitory activity of this segment and represent a mechanism for fine-tuning the activity of *PCSK9* towards the LDLR. GOF mutations in the catalytic domain, which involve charged residues, could affect the positioning of this segment [27].

Another GOF variant G516V (c.1547G > T) was found in five index patients, and cascade screening identified 15 additional carriers. LDL-C levels were higher in those 15 carriers compared with the 27 non-carriers (236 ± 73 versus 124 ± 35 mg/dL; *P* < 0.001). In vitro studies demonstrated the pathogenicity of the G516V variant. Analysing LDL-C differences using generalized estimating equations revealed that the differences between carriers and non-carriers exceeded 39 mg/dL only for the G516V mutation [34].

#### Loss-of-Function Mutations

Contrariwise, genetic *PCSK9* deficiency has been strongly associated with low plasma cholesterol levels and decreased cardiovascular risk, clearly demonstrated from several observational, randomization, and clinical studies [1, 12, 35–40]. Most of the LOF mutations result from either a deficiency in the synthesis or in the secretion of the PCSK9 protein, due to failure to exit the endoplasmic reticulum or failure to undergo the autocatalytic cleavage. Some LOF mutations could dramatically lower plasma PCSK9 up to 79%, and result in no immuno-detectable circulating PCSK9 levels (R104C/V114A, C679*; Y142X/290_292delGCC; Y142X/C679X; Q619X, Q679X, Y142X). However, these null carriers appeared healthy and fertile, with no observable signs of illness due to the absence of PCSK9. PCSK9 null compound heterozygotes did not differ from heterozygotes in any other cardio-metabolic trait, notwithstanding lower median LDL-C [13, 41–44].

Mendelian randomization studies have showed that *PCSK9* genetic variants linked with low LDL-C levels were associated with reduced cardiovascular and all-cause mortality. For instance, the Copenhagen Population Study and Copenhagen City Heart Study have shown a 1 mmol/L (38.7 mg/dL) reduction in LDL-C levels due to the carriage of *PCSK9* variants (R46L, R237W, I474V, and E670G) was associated with a 67% reduction in cardiovascular death (risk ratio 0.33, 95% confidence interval 0.19–0.58; *p* < 0.001) and a 28% reduction in all-cause death (risk ratio 0.72, 95% confidence interval 0.60–0.88; *p* = 0.001) [45].

LOF R46L represents one of the low-frequent *PCSK9* variants (the MAF of the T allele ranged from 1 to 3.2%; 4.8% in a French–Canadian population). Despite its more moderate LDL-lowering effect (9 − 15%), the *PCSK9* R46L allele was associated with a significant reduction in the incidence of CHD (47%) [1]. Homozygous R46L carriers showed a decreased trend of fasting TG levels (115 mg/dL) and higher HDL levels (70 mg/dl). In FH subjects carrying the 46L allele, significantly decreased apo-B and non-HDL concentrations with less severe clinical features were observed [46]. Interestingly, *PCSK9* R46L carriers were observed an allele-dependent lower effect on lipoprotein(a) (Lp(a)) levels versus non-carriers (9 mg/dL in heterozygote, 8 mg/dL in homozygous vs. 10 mg/dL in non-carriers). The application of results regarding the link among PCSK9, LDLR, and Lp(a) metabolism is questionable, especially since Lp(a) plasma levels differ among different ethnic groups [47–49].

There is also evidence that *PCSK9* LOF R46L according to apoE genotype would reveal some metabolic relationships. TG concentrations decreased significantly in the apoE3/E3 carriers with the R46L mutation with invariable plasma free-fatty acid (FFA) concentrations, compared with the apoE3/E2 and apoE3/E4 carriers. Subjects with both the R46L and apoE3/E2 genotypes showed a tendency toward insulin resistance, as indicated by a twofold increase in insulin, and leptin concentrations, compared with those without apoE3/E2 [39]. Emerging evidence suggests that PCSK9 LOF may also influence glucose homeostasis and insulin sensitivity. Beyond cardiovascular benefits, LOF R46L may increase the risk of diabetes in individuals with impaired fasting glucose levels [50]. Furthermore, carriers of *PCSK9* and insertion of insLEU within positions 15 to 21 of the signal peptide of PCSK9 showed also increased occurrence of prediabetes and diabetes status [51]. Another study on animal models showed that glucose clearance is significantly impaired in PCSK9 KO mice fed a standard or a high-fat diet for 20 weeks compared with wild-type animals without affecting insulin sensitivity. PCSK9 KO mice presented larger islets with increased accumulation of cholesteryl esters, paralleled by increased intracellular insulin levels and decreased plasma insulin and C-peptide levels. This phenotype was completely reverted in PCSK9/LDLR DKO mice, implying the LDLR as PCSK9 target responsible for the phenotype observed [52]. Interestingly, results from the genetic and preclinical studies showing an increased risk of diabetes do not extrapolate to randomized trials results, but definitive long-term data are still lacking.

*PCSK9* R46L variant is also associated with a twofold increased prevalence of hepatic steatosis and higher epicardial adiposity in human carriers of the *PCSK9* R46L mutation. A similar observation was recapitulated in *PCSK9* KO mice, showing increased visceral adipose tissue compared to the native genetic form [18, 20].

Postprandial studies have shown that in vivo *PCSK9* deficiency was associated with a twofold decrease in postprandial TG levels [16]. In two heterozygous carriers of LOF *PCSK9* mutation R104C-V114A, no alteration in TG compared with non-carriers in the postprandial state, and also no change in PCSK9 levels following an oral fat load in a small ten-patient sample was present [53]. Another study showed that carriers of LOF variants A53V, I474V, and/or R46L had no differences in PCSK9 levels, significantly lower LDL-C levels, and slightly lower TG levels in the fasting state versus non-carriers. Postprandial, PCSK9 and LDL-C levels are decreased in LOF carriers vs. non-carriers, but TG levels raised significantly to similar levels after the oral fat load. Interestingly, the same author observed that LOF *PCSK9* leads to an inhibitory action on adipocyte differentiation in vitro, in both fasting and postprandial states. Furthermore, PBMC from *PCSK9* LOF variant subjects showed significantly increase mRNA levels of some pro-inflammatory markers after meal [24]. Moderate postprandial lipemia was observed in LOF carriers compared to non-carrier controls after an oral fat load. It is of note that LOF was considered for carriers of the L10ins/A53V and/or I474V and/or R46L mutations, without significant effect on PCSK9 plasma levels. Moreover, as TG, apoB48, and VLDL-C were already lower compared to non-carrier controls (a phenotypic trait not observed in LOF hit variants), it remains to be clarified whether the observed effect should be consistently attributed to *PCSK9* deficiency per se. In any case, there was no significant difference in fasting HDL-C (*p* = 0.46), apoCII (*p* = 0.13), and apoCIII (*p* = 0.66) between the groups, or the CII/CIII ratio between groups, suggesting that alterations in lipolysis are less likely a mechanism for PCSK9’s action on TGRL clearance in studied population [54, 55].

### PCSK9 Inhibitors and Cardiovascular Outcomes

Various approaches have been tried to mimic natural PCSK9 inhibition, such as FDA-approved monoclonal antibodies (mAb) and non-antibody approaches including small interfering RNA (siRNA). Other approaches use therapeutic genome editing via CRISPR/Cas9 technologies (clustered regularly interspaced short palindromic repeats), a base editor which promises prolonged or even permanent reduction in circulating LDL-C levels, small molecule inhibitors (peptides/adnectins), gene silencing using antisense oligonucleotides (ASO), or peptide-based anti-PCSK9 vaccination [56–58]. Recently, the first oral PCSK9 inhibitor, macrocyclic peptide MK-0616, was designed to lower LDL-C via the same biological mechanism as currently approved injectable PCSK9, and it has already demonstrated statistically significant reductions in LDL-C up to 60.9% in phase 2b randomized trial [59]. Moreover, the current pre-clinical evidence regarding novel mechanisms for LDL-C lowering reveals annexin A2 as a natural extrahepatic inhibitor of the PCSK9, while depletion of protein denitrosylase SCoR2 (S-nitroso-coenzyme A reductase 2; AKR1A1) in mice lowers serum cholesterol by inhibiting liver secretion of PCSK9 [60, 61].

Among the therapeutic interventions targeting PCSK9 that are currently in use, PCSK9-mAb evolocumab (Repatha®, Amgen) and alirocumab (Praluent®, Sanofi-Aventis/Regeneron) target circulating PCSK9. This therapy is undoubtedly effective in lowering LDL-C, and results from several large outcomes studies have shown clinical benefit in very high-risk patients, including those with acute coronary syndromes. Treatment with PCSK9 inhibitors decreases LDL-C up to 60%, and favorably affects other lipid parameters such as nonHDL-C, TGs, and Lp(a). More precisely, a 40–50% decrease in apoB, a decrease in non-HDL-C by approximately 50%, and a decrease in Lp (a) by 30% were observed in most studies by positively affecting HDL-C and the level of TAGs. The treatment is very well tolerated and have shown a favorable safety profile (including frequently reported local reaction at the application site) [62]. More recently, inclisiran (LEQVIO®, a siRNA) reported similar lipid-lowering effects by over 50% and maintain its effectduring each the 6-month dosing interval vs. placebo [63].

Extensive evidence of impressive reductions in LDL-C and other lipid parameters has raised questions about whether this reduction translates into a decrease in the cardiovascular events, which is of great clinical interest. Addressing this question, data from clinical trial FOURIER (Further Cardiovascular Outcomes Research with PCSK9 Inhibition in Subjects with Elevated Risk) have demonstrated a significant relative risk reduction of MI (HR 0.73; 95% Cl 0.65 − 0.82) and stroke (HR 0.79; 95% Cl 0.66 − 0.95) [64]. Data from ODYSSEY trial (ODYSSEY Outcomes: Evaluation of Cardiovascular Outcomes After an Acute Coronary Syndrome During Treatment with Alirocumab) showed a reduction and secondary endpoints [62]. Analysis of the inclisiran trial suggests potential benefits for MACE reduction [65]. Further insights on how efficacy of PCSK9 inhibitors may affect CVD outcomes will bring results from currently ongoing cardiovascular outcomes trials, one of evolocumab (VESALIUS-CV [NCT03872401]) and three of inclisiran (ORION-4 [NCT03705234], VICTORION-1 and -2 Prevent [NCT05739383 and NCT05030428, respectively]).

Meta-analyses have evaluated the effects of different PCSK9 modulators compared with control groups. In a meta-analysis, randomizing 3783 patients from ORION clinical trials (1895 to inclisiran and 1888 to placebo), there were no significant reductions in cardiovascular ischemic endpoints with inclisiran in patients with hypercholesterolemia on maximum tolerated statins doses compared with placebo [66].

**Fig. 3:**
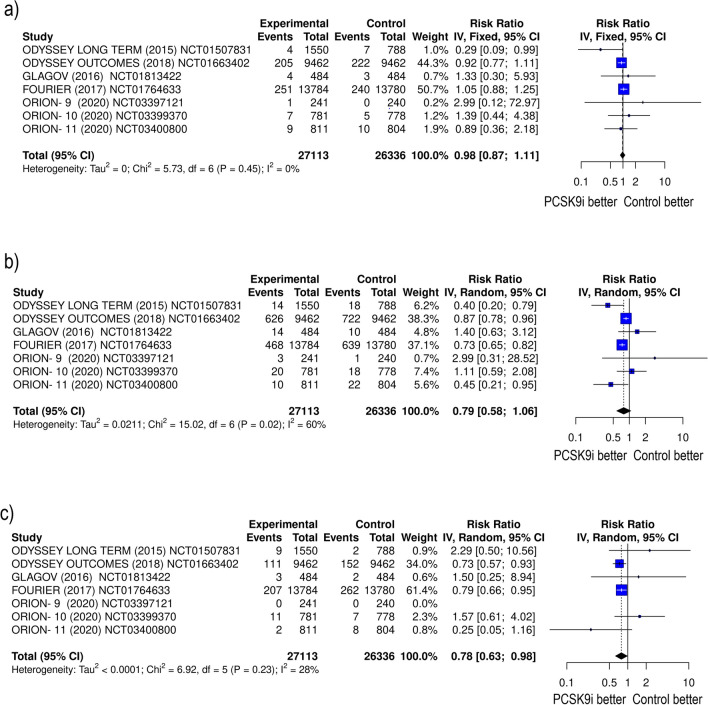
Meta-analysis of randomized clinical trials assessing the effect of different modulators targeting PCSK9 on cardiovascular events (PCSK9-mAb, inclisiran vs. placebo). **a** Forest plot for cardiovascular death. **b** Forest plot treatment for myocardial infarction. **c** Forest plot for stroke. Stroke was significantly reduced following the treatment with a PCSK9 inhibitors (RR 0.78 [95% CI 0.63–0.98] *p* = 0.0352), a similar trend was observed for myocardial infarction (RR 0.79 [95% CI 0.58–1.01] *p* = 0.0951). No significant difference was observed in cardiovascular mortality (RR 0.98 [95% CI 0.86–1.11] *p* = 0.753). Egger’s test for a regression intercept gave a *p*-value of 0.43306 for CVD death, a *p*-value of 0.4905 for MI and *p*-value of 0.2436 for stroke, indicating no evidence of publication bias (Comprehensive Meta-Analysis software V4, Meta-Mar v 3.5.1). The meta-analysis was conducted according to the Preferred Reporting Items for Systematic Reviews and Meta-Analyses (PRISMA) statement. The process of study selection is detailed in Supplementary file 2. Overall estimates of effect were calculated with a fixed or random-effects model and expressed as RR with 95% CI. Statistical significance was set at *p* < 0.05. The assumption of homogeneity between the treatment effects in different trials was tested by *Q* statistic and *I*^2^ statistic. A significant heterogeneity was defined by a *p* < 0.10 at *Q* statistic; *I*^2^ ranging from 0 to 40% might indicate not important heterogeneity, from 30 to 60% might represent moderate heterogeneity, from 50 to 90% might indicate substantial heterogeneity, and from 75 to 100% might represent considerable heterogeneity. Abbreviations: CI, confidence interval. The black square represents the weight of each study, the canter of the square represents the risk ratio (RR), and the length of each line around the point represents its 95% confidence interval (95% CI). The overall summary estimate (a result of the meta-analysis) is represented by a lozenge (diamond shape) at the end of the graph

PCSK9-mAb significantly reduced the risk of stroke (RR 0.75; 95% CI 0.66–0.86, *p* < 0.0001) and MI (RR 0.81; 95% CI 0.76–0.87, *p* < 0.00001), but not the risk of cardiovascular death (RR 0.96; 95% CI 0.86–1.07, *p* = 0.45) compared with placebo [67]. Similarly, no significant reduction of all-cause mortality was found (RR 0.88; 95% CI 0.72–1.07; *p* = 0.182) in meta-analysis of 25 randomized controlled trials (RCTs) comparing PCSK9 mAbs alirocumab and evolocumab with placebos or active drugs in patients at high cardiovascular risk. Both alirocumab (RR 0.89; 95% CI 0.83–0.95; *p* < 0.001) and evolocumab (RR 0.86; 95% CI 0.80–0.92; *p* < 0.001) were associated with a lower risk of major cardiovascular events (MACEs), especially in secondary prevention (alirocumab group: RR 0.88; 95% CI 0.82–0.95; *p* < 0.001; evolocumab group: RR 0.86; 95% CI 0.80–0.92; *p* < 0.001). The reduction in MACEs was observed in Caucasians but not in Asian subjects [68]. Interestingly, the meta-analysis of clinical outcomes of PCSK9 modulators (evolocumab, alirocumab, inclisiran) in patients with established atherosclerotic cardiovascular disease (ASCVD) indicated a reduction in the composite outcomes of MI, stroke, and cardiovascular death (relative risk RR 0.80, 95% CI 0.73–0.87) and MI, stroke, unstable angina (requiring revascularization), and cardiovascular death (RR 0.85, 95% CI 0.74–0.97). However, individual effects on mortality, cardiovascular death, MI, and stroke remained nonsignificant [69]. Another finding from a metanalytic study of nine trials, including a total of 54,301 participants was that both alirocumab and evolocumab, were associated with reductions in MI (RR 0.86; 95% CI 0.77–0.95 and RR 0.73; 95% CI 0.65–0.82 respectively) and stroke (RR 0.76; 95% CI 0.60–0.96 and RR 0.79; 95% CI 0.66–0.94 respectively). Moreover, the use of alirocumab was associated with reductions in all-cause mortality compared with control (RR 0.83; 95% CI 0.72–0.95), while evolocumab was associated with increased all-cause mortality compared with alirocumab (RR 1.26, 95% CI 1.04–1.52) [70]. An earlier meta-analytic study of 67 RCTs including 259,429 participants with PCSK9 inhibitors plus statin confirmed the significantly reduced risk of non-fatal MI (RR 0.82; 95% CI 0.72–0.93, *p* = 0.003) or stroke (RR 0.74; 95% CI 0.65–0.85, *p* < 0.001) [71]. The results of the most recent study of Imran et al. align with overall previous findings in MACE. Moreover, alirocumab reduced all-cause mortality, which is likely due to the inclusion of new randomized trials that have been published since the previous studies were conducted, and longer follow-up periods [72]. In conclusion, the investigation into the efficacy of pharmacologic agents targeting PCSK9 suggests an effect rather for lowering the risk of MI and stroke than for cardiovascular mortality (Fig. 3).

Post hoc and secondary analysis of long-term studies indicate greater clinical benefits in subgroups of patients with a heavier burden of atherosclerosis or complex plaque morphology, with more recent ischemic events, or in those who reach very low LDL levels.

Safety of living with very low levels of LDL-C concentrations achieved (30 mg/dL with the evolocumab; < 15 mg/dL with the alirocumab) was reported as well as benefits in reducing the risk of angina, MI, or cerebrovascular disorder and total mortality [64, 73]. Patients closer to the most recent MI, a history of multiple MIs, or multivessel coronary artery disease gained greater absolute risk reductions (3.5 to 1%) from evolocumab [74]. Furthermore, evolocumab reduced the risk of MI related to plaque rupture [75]. The risk of ischemic stroke was significantly reduced by evolocumab (1.2 vs. 1.6%), in subgroup analyses of patients with prior ischemic stroke vs. patients without [76]. In the ODYSSEY OUTCOMES subanalysis, the estimated absolute risk reductions with alirocumab were numerically greater in patients with previous MI (MACE, 1.91% vs. 1.42%; death, 1.35% vs. 0.41%) [77]. A further post hoc analysis evaluating the efficacy of alirocumab according to metabolic risk factors concluded consistently reduced MACE across categories defined by the number of metabolic risk factors, but absolute risk reduction (aRR) increased with the number of metabolic risk factors findings revealed larger aRR in cardiovascular events compared to individuals without diabetes (7.7–14.6%) and metabolic syndrome (0.91 to 3.82%) [78]. Results are in concordance with post hoc analyses from the FOURIER trial (patients with atherosclerotic cardiovascular disease and metabolic syndrome, MetS). Evolocumab reduced the risk of CVD events in patients with MetS (evolocumab vs. placebo [HR 0.83, 95% CI 0.76–0.91]), patients with vs. without MetS [HR 0.89, 95% CI 0.79–1.01] *p*_interaction_ = 0.39) [79]. PCSK9 mAb appears to be effective in reducing the risk of MACE by 18% (OR 0.82, 95% CI 0.74–0.90) in subjects with diabetes and dyslipidemia [80].

Moreover, in patients with MetS, evolocumab and alirocumab did not result in an increased risk of developing new-onset diabetes (NOD), or worsening glycemia [78, 81]. The use of PCSK9 inhibitors raised concerns about the risk of NOD, especially taking into consideration results from genetic studies. Safety reports from RCTs showed that evolocumab and alirocumab were primarily related to mild hyperglycemia rather than diabetes, with most of the hyperglycaemic events occurring during the first 6 months of treatment [82]. This effect disappears after drug withdrawal and treatment with PCSK9 inhibitors should be of minimal concern [83]. A meta-analysis by Khan et al. did not show any significant association between PCSK9 inhibitors and NOD (HR 1.00, 95% CI 0.93–1.07; *p* = 0.96) [84]. A recent meta-analysis of 39 randomized clinical trials including 35,896 participants treated with alirocumab or evolocumab did not find any association between the use of these drugs and NOD (*p* = 0.97) [85]. Finally, besides effects on lipids and traditional cardiovascular outcomes, a recent post hoc analysis highlights the positive effects of both PCSK9-mAb on venous thromboembolic events (31% relative risk reduction) and aortic stenosis progression [86, 87] (Table 1).

**Table 1:** Overview of post hoc analyses and meta-analyses involving PCSK9 inhibitors

Inhibition strategy	Study population	Control	Reported effect	References
PCSK9 mAb Alirocumab	ODYSSEY OUTOMES – propensity score-matching analysis	Placebo	• MACE rates in alirocumab group vs. placebo: 2.3 vs. 3.2 events per 100 patient-year(s) (*p* = 0.047); all-cause mortality: 0.7% vs. 1.2% (*p* = 0.06); MI 1.5% vs. 2.1% (*p* = 0.11)	[73]
PCSK9 mAb Alirocumab	ODYSSEY OUTOMES – history of previous MI	Placebo/patients without a previous MI	• MACE reduction 1.91% vs. 1.42% • death reduction 1.35% vs. 0.41%	[77]
PCSK9 mAb Alirocumab	ODYSSEY OUTOMES –metabolic risk factor stratification	Placebo	• MACE incidence increased with each metabolic risk factor from 7.8% (no risk factors) to 19.6% (five risk factors; HR 1.18, 95% CI 1.13–1.24 per metabolic risk factor) • Decreased relative risk of MACE consistently across categories defined by the number of metabolic risk factors (*p*_interaction_ = 0.77); absolute risk reduction (aRR) increased with the number of metabolic risk factors (no risk factors aRR 0.7%, − 1.81 to 3.29 vs. five risk factors aRR 3.9%, − 1.45 to 9.25; *p*_interaction_ < 0.001) • In no diabetes patients-incidence of MACE increased from 7.7% in patients with no metabolic risk factors to 14.6% in those with five metabolic risk factors; aRR with alirocumab increased from 0.91% in patients with no metabolic risk factors to 3.82% in those with five factors	[78]
PCSK9 mAb Evolocumab	FOURIER trial: high-risk subgroups with recent MI, a history of multiple MIs, or multivessel coronary artery disease	Patients without a recent MI, a history of multiple MIs, or multivessel coronary artery disease	• CVD events relative risk reduction in high-risk subgroups exceed up to 21% up to 8% in those without CVD events absolute risk reduction in high-risk subgroups exceed 3% to 1% in those without	[74]
PCSK9 mAb Evolocumab	FOURIER trial: Cerebrovascular events Patients stratified by prior stroke	Placebo Patients without a prior ischemic stroke	• Significant reduction in all strokes (1.5% versus 1.9%; HR 0.79 [95% CI 0.66–0.95]; *p* = 0.01); ischemic stroke (1.2% versus 1.6%; HR 0.75 [95% CI 0.62–0.92]; *p* = 0.005); no difference in hemorrhagic stroke (0.21% versus 0.18%; HR 1.16 [95% CI 0.68–1.98]; *p* = 0.59) • Patients with prior ischemic stroke HR (95% CIs) were 0.85 (0.72–1.00) for the cardiovascular composite, 0.90 (0.68–1.19) for all strokes, and 0.92 (0.68–1.25) for ischemic stroke (*p*_interactions_, 0.91, 0.22, and 0.09, respectively)	[76]
PCSK9 mAb Evolocumab	FOURIER trial: patients with peripheral artery disease	Patients without peripheral artery disease	• Greater absolute benefit in reducing composite of cardiovascular death, myocardial infarction, stroke, hospital admission for unstable angina, or coronary revascularization (absolute risk reduction 3.5 vs. 1.6%)	[88]
PCSK9 mAb Evolocumab	FOURIER trial: patients were stratified based on the National Cholesterol Education Program Adult Treatment Panel III MetS criteria; in secondary analyses, patients were further sub stratified by diabetes at baseline	Placebo	• Reduced risk of CVD events in patients with MetS (evolocumab vs. placebo (HR 0.83, 95% CI 0.76–0.91) • Reduced risk of CVD events in patients with vs. without MetS (HR 0.89, 95% CI 0.79–1.01] *p*_interaction_ = 0.39)	[79]
PCSK9 mAbs Alirocumab Evolocumab	32 randomized controlled trials (21 of statins, 12 of PCSK9 inhibitors	Less intensive therapy corresponded to an active control group/placebo	• Not associated with incident DM (RR 1.00; 95% CI 0.93–1.07; *p* = 0.96; *p*_interaction_ = 0.02)	[84]
PCSK9 mAbs Alirocumab Evolocumab	8 randomized control trials enrolling 20 651 patients with hypercholesterolemia and diabetes	Placebo	• Reducing risk of MACE (OR 0.82, 95% CI 0.74–0.90)	[80]
PCSK9 siRNA Inclisiran	Pooled analysis ORION-9, -10, -11	Placebo	• No significant reductions in cardiovascular ischemic endpoints	[66]
PCSK9 siRNA Inclisiran	Patient-level analysis of phase III trials, pooled analysis of ORION-9, -10, -11, included patients with heterozygous familial hypercholesterolemia, ASCVD, or ASCVD risk equivalent on maximally tolerated statin-therapy	Placebo	• Significant reduction composite MACE (OR 0.74, 95% CI 0.58–0.94) • Not reduction of not fatal and non-fatal MIs (OR 0.80, 95% CI 0.50–1.27) or fatal and non-fatal stroke (OR 0.86, 95% CI 0.41–1.81)	[65]

*CI* confidence interval, *RR* risk ratio, *aRR* absolute risk reduction, *OR* odds ratio, *HR* hazard ratio, *MACE* major adverse cardiovascular events, *ASCVD* atherosclerotic cardiovascular disease, *CVD* cardiovascular disease, *MI* myocardial infarction

## Conclusions

Genetic studies have clearly demonstrated that *PCSK9* is a major determinant of cholesterol homeostasis, and several GOF and LOF *PCSK9* mutations have been described, highlighting a key role for *PCSK9* in regulating lipid metabolism. In this regard, *PSCK9*-targeting agents have become the most promising therapeutic approach to manage hypercholesterolemia and related diseases and reduce the risk of some cardiovascular outcomes. However, many challenging issues related to *PCSK9* still exist and a better understanding of the genetic variations in *PCSK9* may clarify its biologic role, especially as a promising tool for novel lipid-lowering therapies.

## Supplementary Information

Below is the link to the electronic supplementary material.

Supplementary file1 (DOCX 33 kb)

Supplementary file2 (PPTX 43 kb)

## References

[1] Cohen JC, Boerwinkle E, Mosley TH Jr, Hobbs HH. Sequence variations in PCSK9, low LDL, and protection against coronary heart disease. N Engl J Med. 2006;354(12):1264–72. 10.1056/NEJMoa054013. 16554528 10.1056/NEJMoa054013

[2] Lepor NE, Kereiakes DJ. The PCSK9 inhibitors: a novel therapeutic target enters clinical practice. Am Health Drug Benefits. 2015;8(9):483–9. 26834934 PMC4719137

[3] Ward NC, Page MM, Watts GF. PCSK9 inhibition riding a new wave of coronary prevention. Clin Sci (Lond). 2019;133(2):205–24. 10.1042/CS20171300. 30670671 10.1042/CS20171300

[4] Abifadel M, Varret M, Rabès JP, et al. Mutations in PCSK9 cause autosomal dominant hypercholesterolemia. Nat Genet. 2003;34(2):154–6. 10.1038/ng1161. 12730697 10.1038/ng1161

[5] Benjannet S, Rhainds D, Essalmani R, et al. NARC-1/PCSK9 and its natural mutants: zymogen cleavage and effects on the low density lipoprotein (LDL) receptor and LDL cholesterol. J Biol Chem. 2004;279(47):48865–75. 10.1074/jbc.M409699200. 15358785 10.1074/jbc.M409699200

[6] Chaudhary R, Garg J, Shah N, Sumner A. PCSK9 inhibitors: a new era of lipid lowering therapy. World J Cardiol. 2017;9(2):76–91. 10.4330/wjc.v9.i2.76. 28289523 10.4330/wjc.v9.i2.76 PMC5329749

[7] Norata GD, Tavori H, Pirillo A, Fazio S, Catapano AL. Biology of proprotein convertase subtilisin kexin 9: beyond low-density lipoprotein cholesterol lowering. Cardiovasc Res. 2016;112(1):429–42. 10.1093/cvr/cvw194. 27496869 10.1093/cvr/cvw194 PMC5031950

[8] Essalmani R, Susan-Resiga D, Chamberland A, et al. In vivo evidence that furin from hepatocytes inactivates PCSK9. J Biol Chem. 2011;286(6):4257–63. 10.1074/jbc.M110.192104. 21147780 10.1074/jbc.M110.192104 PMC3039354

[9] Leren TP. Sorting an LDL receptor with bound PCSK9 to intracellular degradation. Atherosclerosis. 2014;237:76–81. 10.1016/j.atherosclerosis.2014.08.038. 25222343 10.1016/j.atherosclerosis.2014.08.038

[10] Ahn CH, Choi SH. New drugs for treating dyslipidemia: beyond statins. Diabetes Metab J. 2015;39(2):87–94. 10.4093/dmj.2015.39.2.87. (Adapted with permission of Copyright © 2021 Korean Diabetes Association from Diabetes Metab J. 2015;39:87-94 Reprinted with permission from The Korean Diabetes Association). 25922802 10.4093/dmj.2015.39.2.87 PMC4411552

[11] Martin FJ, Amode MR, Aneja A, et al. Ensembl 2023. Nucleic Acids Res. 2023;51(D1):D933-d941. 10.1093/nar/gkac958. 36318249 10.1093/nar/gkac958 PMC9825606

[12] Qiu C, Zeng P, Li X, et al. What is the impact of PCSK9 rs505151 and rs11591147 polymorphisms on serum lipids level and cardiovascular risk: a meta-analysis. Lipids Health Dis. 2017;16(1):111. 10.1186/s12944-017-0506-6. 28606094 10.1186/s12944-017-0506-6 PMC5469167

[13] Benjannet S, Hamelin J, Chrétien M, Seidah NG. Loss- and gain-of-function PCSK9 variants: cleavage specificity, dominant negative effects, and low density lipoprotein receptor (LDLR) degradation. J Biol Chem. 2012;287(40):33745–55. 10.1074/jbc.M112.399725. 22875854 10.1074/jbc.M112.399725 PMC3460471

[14] Abifadel M, Guerin M, Benjannet S, et al. Identification and characterization of new gain-of-function mutations in the PCSK9 gene responsible for autosomal dominant hypercholesterolemia. Atherosclerosis. 2012;223(2):394–400. 10.1016/j.atherosclerosis.2012.04.006. 22683120 10.1016/j.atherosclerosis.2012.04.006

[15] De Castro-Orós I, Pocoví M, Civeira F. The genetic basis of familial hypercholesterolemia: inheritance, linkage, and mutations. Appl Clin Genet. 2010;3:53–64. 10.2147/TACG.S8285. 23776352 10.2147/tacg.s8285 PMC3681164

[16] Le May C, Kourimate S, Langhi C, et al. Proprotein convertase subtilisin kexin type 9 null mice are protected from postprandial triglyceridemia. Arterioscler Thromb Vasc Biol. 2009;29(5):684–90. 10.1161/ATVBAHA.108.181586. 19265033 10.1161/ATVBAHA.108.181586

[17] Gagnon A, Ooi TC, Cousins M, et al. The anti-adipogenic effect of peripheral blood mononuclear cells is absent with PCSK9 loss-of-function variants. Obesity (Silver Spring). 2016;24(11):2384–91. 10.1002/oby.21656. 27662822 10.1002/oby.21656

[18] Baragetti A, Balzarotti G, Grigore L, et al. PCSK9 deficiency results in increased ectopic fat accumulation in experimental models and in humans. Eur J Prev Cardiol. 2017;24(17):1870–7. 10.1177/2047487317724342. 28758421 10.1177/2047487317724342

[19] Lagace TA. PCSK9 and LDLR degradation: regulatory mechanisms in circulation and in cells. Curr Opin Lipidol. 2014;25(5):387–93. 10.1097/mol.0000000000000114. 25110901 10.1097/MOL.0000000000000114 PMC4166010

[20] Baragetti A, Grejtakova D, Casula M, et al. Proprotein convertase subtilisin-kexin type-9 (PCSK9) and triglyceride-rich lipoprotein metabolism: facts and gaps. Pharmacol Res. 2018;130:1–11. 10.1016/j.phrs.2018.01.025. 29428206 10.1016/j.phrs.2018.01.025

[21] Leren TP. Mutations in the PCSK9 gene in Norwegian subjects with autosomal dominant hypercholesterolemia. Clin Genet. 2004;65(5):419–22. 10.1111/j.0009-9163.2004.0238.x. 15099351 10.1111/j.0009-9163.2004.0238.x

[22] Allard D, Amsellem S, Abifadel M, et al. Novel mutations of the PCSK9 gene cause variable phenotype of autosomal dominant hypercholesterolemia. Hum Mutat. 2005;26(5):497. 10.1002/humu.9383. 16211558 10.1002/humu.9383

[23] Noguchi T, Katsuda S, Kawashiri MA, et al. The E32K variant of PCSK9 exacerbates the phenotype of familial hypercholesterolaemia by increasing PCSK9 function and concentration in the circulation. Atherosclerosis. 2010;210(1):166–72. 10.1016/j.atherosclerosis.2009.11.018. 20006333 10.1016/j.atherosclerosis.2009.11.018

[24] Cameron J, Holla OL, Laerdahl JK, et al. Characterization of novel mutations in the catalytic domain of the PCSK9 gene. J Intern Med. 2008;263(4):420–31. 10.1111/j.1365-2796.2007.01915.x. 18266662 10.1111/j.1365-2796.2007.01915.x

[25] Bourbon M, Alves AC, Medeiros AM, Silva S, Soutar AK, Investigators of Portuguese FH Study. Familial hypercholesterolaemia in Portugal. Atherosclerosis. 2008;196(2):633–42. 10.1016/j.atherosclerosis.2007.07.019. 17765246 10.1016/j.atherosclerosis.2007.07.019

[26] Pisciotta L, Priore Oliva C, Cefalù AB, et al. Additive effect of mutations in LDLR and PCSK9 genes on the phenotype of familial hypercholesterolemia. Atherosclerosis. 2006;186(2):433–40. 10.1016/j.atherosclerosis.2005.08.015. 16183066 10.1016/j.atherosclerosis.2005.08.015

[27] Wierød L, Cameron J, Strøm TB, Leren TP. Studies of the autoinhibitory segment comprising residues 31–60 of the prodomain of PCSK9: possible implications for the mechanism underlying gain-of-function mutations. Mol Genet Metab Rep. 2016;9:86–93. 10.1016/j.ymgmr.2016.11.003. 27896130 10.1016/j.ymgmr.2016.11.003 PMC5121147

[28] Benjannet S, Rhainds D, Hamelin J, Nassoury N, Seidah NG. The proprotein convertase (PC) PCSK9 is inactivated by furin and/or PC5/6A: functional consequences of natural mutations and post-translational modifications. J Biol Chem. 2006;281(41):30561–72. 10.1074/jbc.M606495200. 16912035 10.1074/jbc.M606495200

[29] Abifadel M, Elbitar S, El Khoury P, et al. Living the PCSK9 adventure: from the identification of a new gene in familial hypercholesterolemia towards a potential new class of anticholesterol drugs. Curr Atheroscler Rep. 2014;16(9):439. 10.1007/s11883-0140439-8. 25052769 10.1007/s11883-014-0439-8

[30] Ouguerram K, Chetiveaux M, Zair Y, et al. Apolipoprotein B100 metabolism in autosomal-dominant hypercholesterolemia related to mutations in PCSK9. Arterioscler Thromb Vasc Biol. 2004;24(8):1448–53. 10.1161/01.ATV.0000133684.77013.88. 15166014 10.1161/01.ATV.0000133684.77013.88

[31] Ding K, Kullo IJ. Molecular population genetics of PCSK9: a signature of recent positive selection. Pharmacogenet Genomics. 2008;18(3):169–79. 10.1097/fpc.0b013e3282f44d99. 18300938 10.1097/FPC.0b013e3282f44d99 PMC2842919

[32] Aung LH, Yin RX, Wu DF, Cao XL, Hu XJ, Miao L. Proprotein convertase subtilisin/kexin type 9 gene E670G polymorphism interacts with alcohol consumption to modulate serum lipid levels. Int J Med Sci. 2011;10(2): 124–132. https://www.medsci.org/v10p0124.htm. Accessed 09.11.2023. 10.7150/ijms.5296 PMC3547209 23329883

[33] Mayne J, Ooi TC, Raymond A, et al. Differential effects of PCSK9 loss of function variants on serum lipid and PCSK9 levels in Caucasian and African Canadian populations. Lipids Health Dis. 2013;12:70. 10.1186/1476-511X-12-70. 23663650 10.1186/1476-511X-12-70 PMC3661383

[34] Huijgen R, Blom DJ, Hartgers ML, et al. Novel PCSK9 (proprotein convertase subtilisin kexin type 9) variants in patients with familial hypercholesterolemia from Cape Town. Arterioscler Thromb Vasc Biol. 2021;41(2):934–43. 10.1161/ATVBAHA.120.314482. (Erratum in: Arterioscler Thromb Vasc Biol. 2021 Jan;41(1):e77). 33147992 10.1161/ATVBAHA.120.314482

[35] Cameron J, Holla ØL, Ranheim T, Kulseth MA, Berge KE, Leren TP. Effect of mutations in the PCSK9 gene on the cell surface LDL receptors. Hum Mol Genet. 15(9): 1551–1558. 10.1093/hmg/ddl077. 10.1093/hmg/ddl077 16571601

[36] Cohen J, Pertsemlidis A, Kotowski IK, Graham R, Garcia CK, Hobbs HH. Low LDL cholesterol in individuals of African descent resulting from frequent nonsense mutations in PCSK9. Nat Genet. 2005;37(2):161–5. 10.1038/ng1509. (Erratum in: Nat Genet. 2005 Mar;37(3):328). 15654334 10.1038/ng1509

[37] Humphries SE, Neely RD, Whittall RA, et al. Healthy individuals carrying the PCSK9 p.R46L variant and familial hypercholesterolemia patients carrying PCSK9 p.D374Y exhibit lower plasma concentrations of PCSK9. Clin Chem. 2009;55(12):2153–61. 10.1373/clinchem.2009.129759. 19797716 10.1373/clinchem.2009.129759

[38] Benn M, Nordestgaard BG, Grande P, Schnohr P, Tybjaerg-Hansen A. PCSK9 R46L, low-density lipoprotein cholesterol levels, and risk of ischemic heart disease: 3 independent studies and meta-analyses. J Am Coll Cardiol. 2010;55(25):2833–42. 10.1016/j.jacc.2010.02.044. 20579540 10.1016/j.jacc.2010.02.044

[39] Awan Z, Delvin EE, Levy E, et al. Regional distribution and metabolic effect of PCSK9 insLEU and R46L gene mutations and apoE genotype. Can J Cardiol. 2013;29(8):927–33. 10.1016/j.cjca.2013.03.004. 23743349 10.1016/j.cjca.2013.03.004

[40] Tarasov K, Ekroos K, Suoniemi M, et al. Molecular lipids identify cardiovascular risk and are efficiently lowered by simvastatin and PCSK9 deficiency. J Clin Endocrinol Metab. 2014;1:E45-52. 10.1210/jc.2013-2559. 10.1210/jc.2013-2559 PMC3928964 24243630

[41] Zhao Z, Tuakli-Wosornu Y, Lagace TA, et al. Molecular characterization of loss-of-function mutations in PCSK9 and identification of a compound heterozygote. Am J Hum Genet. 2006;79(3):514–23. 10.1086/507488. 16909389 10.1086/507488 PMC1559532

[42] Cariou B, Ouguerram K, Zaïr Y, et al. PCSK9 dominant negative mutant results in increased LDL catabolic rate and familial hypobetalipoproteinemia. Arterioscler Thromb Vasc Biol. 2009;29(12):2191–7. 10.1161/ATVBAHA.109.194191. 19762784 10.1161/ATVBAHA.109.194191

[43] Wu NQ, Li JJ. PCSK9 gene mutations and low-density lipoprotein cholesterol. Clin Chim Acta. 2014;431:148–53. 10.1016/j.cca.2014.01.043. 24518357 10.1016/j.cca.2014.01.043

[44] Peloso GM, Lange LA, Varga TV, et al. Association of exome sequences with cardiovascular traits among blacks in the Jackson Heart Study. Circ Cardiovasc Genet. 2016;9(4):368–74. 10.1161/CIRCGENETICS.116.001410. 27422940 10.1161/CIRCGENETICS.116.001410 PMC4988917

[45] Benn M, Tybjærg-Hansen A, Nordestgaard BG. Low LDL cholesterol by PCSK9 variation reduces cardiovascular mortality. J Am Coll Cardiol. 2019;73(24):3102–14. 10.1016/j.jacc.2019.03.517. 31221259 10.1016/j.jacc.2019.03.517

[46] Saavedra YG, Dufour R, Davignon J, Baass A. PCSK9 R46L, lower LDL, and cardiovascular disease risk in familial hypercholesterolemia: a cross-sectional cohort study. Arterioscler Thromb Vasc Biol. 2014;34(12):2700–5. 10.1161/ATVBAHA.114.304406. 25278291 10.1161/ATVBAHA.114.304406

[47] Nordestgaard BG, Chapman MJ, Ray K, European Atherosclerosis Society Consensus Panel, et al. Lipoprotein(a) as a cardiovascular risk factor: current status. Eur Heart J. 2010;31(23):2844–53. 10.1093/eurheartj/ehq386. 20965889 10.1093/eurheartj/ehq386 PMC3295201

[48] Kronenberg F. Lipoprotein(a): from causality to treatment. Curr Atheroscler Rep. 2024;26(3):75–82. 10.1007/s11883-024-01187-6. 38252372 10.1007/s11883-024-01187-6 PMC10881767

[49] Langsted A, Nordestgaard BG, Benn M, Tybjærg-Hansen A, Kamstrup PR. PCSK9 R46L loss-of-function mutation reduces lipoprotein(a), LDL cholesterol, and risk of aortic valve stenosis. J Clin Endocrinol Metab. 2016;101(9):3281–370. 10.1210/jc.2016-1206. 27218270 10.1210/jc.2016-1206

[50] Ference BA, Robinson JG, Brook RD, et al. Variation in PCSK9 and HMGCR and risk of cardiovascular disease and diabetes. N Engl J Med. 2016;375(22):2144–53. 10.1056/nejmoa1604304. 27959767 10.1056/NEJMoa1604304

[51] Ferri N, Ruscica M. Proprotein convertase subtilisin/kexin type 9 (PCSK9) and metabolic syndrome: insights on insulin resistance, inflammation, and atherogenic dyslipidemia. Endocrine. 2016;54(3):588–601. 10.1007/s12020-016-0939-0. 27038318 10.1007/s12020-016-0939-0

[52] Da Dalt L, Ruscica M, Bonacina F, et al. PCSK9 deficiency reduces insulin secretion and promotes glucose intolerance: the role of the low-density lipoprotein receptor. Eur Heart J. 2019;40(4):357–68. 10.1093/eurheartj/ehy357. 29982592 10.1093/eurheartj/ehy357

[53] Cariou B, Langhi C, Le Bras M, et al. Plasma PCSK9 concentrations during an oral fat load and after short term high-fat, high-fat high-protein and high-fructose diets. Nutr Metab (Lond). 2013;10(1):4. 10.1186/1743-7075-10-4. 23298392 10.1186/1743-7075-10-4 PMC3548771

[54] Ooi TC, Krysa JA, Chaker S, et al. The effect of PCSK9 loss-of-function variants on the postprandial lipid and ApoB-lipoprotein response. J Clin Endocrinol Metab. 2017;102(9):3452–60. 10.1210/jc.2017-00684. 28673045 10.1210/jc.2017-00684

[55] Verbeek R, Boyer M, Boekholdt SM, et al. Carriers of the PCSK9 R46L variant are characterized by an antiatherogenic lipoprotein profile assessed by nuclear magnetic resonance spectroscopy-brief report. Arterioscler Thromb Vasc Biol. 2017;37(1):43–8. 10.1161/ATVBAHA.116.307995. 27856457 10.1161/ATVBAHA.116.307995 PMC6284798

[56] Musunuru K, Chadwick AC, Mizoguchi T, et al. In vivo CRISPR base editing of PCSK9 durably lowers cholesterol in primates. Nature. 2021;593(7859):429–34. 10.1038/s41586-021-03534-y. 34012082 10.1038/s41586-021-03534-y

[57] Rothgangl T, Dennis MK, Lin PJC, et al. In vivo adenine base editing of PCSK9 in macaques reduces LDL cholesterol levels. Nat Biotechnol. 2021;39(8):949–57. 10.1038/s41587-021-00933-4. 34012094 10.1038/s41587-021-00933-4 PMC8352781

[58] Wierzbicki AS, Viljoen A. Anti-sense oligonucleotide therapies for the treatment of hyperlipidaemia. Expert Opin Biol Ther. 2016;16(9):1125–34. 10.1080/14712598.2016.1196182. 27248482 10.1080/14712598.2016.1196182

[59] Ballantyne CM, Banka P, Mendez G, et al. Phase 2b randomized trial of the oral PCSK9 inhibitor MK-0616. J Am Coll Cardiol. 2023;81(16):1553–64. 10.1016/j.jacc.2023.02.018. 36889610 10.1016/j.jacc.2023.02.018

[60] Seidah NG, Poirier S, Denis M, et al. Annexin A2 is a natural extrahepatic inhibitor of the PCSK9-induced LDL receptor degradation. PLoS ONE. 2012;7(7):e41865. 10.1371/journal.pone.0041865. 22848640 10.1371/journal.pone.0041865 PMC3407131

[61] Stomberski CT, Venetos NM, Zhou HL, et al. A multienzyme S-nitrosylation cascade regulates cholesterol homeostasis. Cell Rep. 2022;41(4):111538. 10.1016/j.celrep.2022.111538. 36288700 10.1016/j.celrep.2022.111538 PMC9667709

[62] Schwartz GG, Steg PG, Szarek M, ODYSSEY OUTCOMES Committees and Investigators, et al. Alirocumab and cardiovascular outcomes after acute coronary syndrome. N Engl J Med. 2018;379(22):2097–107. 10.1056/nejmoa1801174. 30403574 10.1056/NEJMoa1801174

[63] Ray KK, Kallend D, Leiter L, et al. Effect of inclisiran on LDL-C reduction across different background lipid lowering treatments: analyses from ORION-11. J Am Coll Cardiol. 2020;75(11):2078. 10.1016/S0735-1097(20)32705-4.

[64] Sabatine MS, Giugliano RP, Keech AC, FOURIER Steering Committee and Investigators, et al. Evolocumab and clinical outcomes in patients with cardiovascular disease. N Engl J Med. 2017;376(18):1713–22. 10.1056/nejmoa1615664. 28304224 10.1056/NEJMoa1615664

[65] Ray KK, Raal FJ, Kallend DG, ORION Phase III investigators, et al. Inclisiran and cardiovascular events: a patient-level analysis of phase III trials. Eur Heart J. 2023;44(2):129–38. 10.1093/eurheartj/ehac594. 36331326 10.1093/eurheartj/ehac594 PMC9825807

[66] Asbeutah AAA, Asbeutah SA, Abu-Assi MA. A meta-analysis of cardiovascular outcomes in patients with hypercholesterolemia treated with inclisiran. Am J Cardiol. 2020;128:218–9. 10.1016/j.amjcard.2020.05.024. 32482309 10.1016/j.amjcard.2020.05.024

[67] Ma W, Guo X, Ma Y, Hu Z. Meta-analysis of randomized clinical trials comparing PCSK9 monoclonal antibody versus ezetimibe/placebo in patients at high cardiovascular risk. Atherosclerosis. 2021;326:25–34. 10.1016/j.atherosclerosis.2021.04.008. 34004550 10.1016/j.atherosclerosis.2021.04.008

[68] Mu G, Xiang Q, Zhou S, et al. Efficacy and safety of PCSK9 monoclonal antibodies in patients at high cardiovascular risk: an updated systematic review and meta-analysis of 32 randomized controlled trials. Adv Ther. 2020;37(4):1496–521. 10.1007/s12325-020-01259-4. 32108309 10.1007/s12325-020-01259-4

[69] Talasaz AH, Ho AJ, Bhatty F, et al. Meta-analysis of clinical outcomes of PCSK9 modulators in patients with established ASCVD. Pharmacotherapy. 2021;41(12):1009–23. 10.1002/phar.2635. 34657313 10.1002/phar.2635

[70] Wang X, Wen D, Chen Y, Ma L, You C. PCSK9 inhibitors for secondary prevention in patients with cardiovascular diseases: a Bayesian network meta-analysis. Cardiovasc Diabetol. 2022;21(1):107. 10.1186/s12933-022-01542-4. 35706032 10.1186/s12933-022-01542-4 PMC9202167

[71] Chaiyasothi T, Nathisuwan S, Dilokthornsakul P, et al. Effects of non-statin lipid-modifying agents on cardiovascular morbidity and mortality among statin-treated patients: a systematic review and network meta-analysis. Front Pharmacol. 2019;10:547. 10.3389/fphar.2019.00547. 31191304 10.3389/fphar.2019.00547 PMC6540916

[72] Imran TF, Khan AA, Has P, et al. Proprotein convertase subtilisn/kexin type 9 inhibitors and small interfering RNA therapy for cardiovascular risk reduction: a systematic review and meta-analysis. PLoS ONE. 2023;18(12):e0295359. 10.1371/journal.pone.0295359. 38055686 10.1371/journal.pone.0295359 PMC10699593

[73] Schwartz GG, Szarek M, Bhatt DL, ODYSSEY OUTCOMES Investigators, et al. Transiently achieved very low LDL-cholesterol levels by statin and alirocumab after acute coronary syndrome are associated with cardiovascular risk reduction: the ODYSSEY OUTCOMES trial. Eur Heart J. 2023;44(16):1408–17. 10.1093/eurheartj/ehad144. 36879424 10.1093/eurheartj/ehad144 PMC10119028

[74] Sabatine MS, De Ferrari GM, Giugliano RP, et al. Clinical benefit of evolocumab by severity and extent of coronary artery disease: analysis from FOURIER. Circulation. 2018;138(8):756–66. 10.1161/CIRCULATIONAHA.118.034309. 29626068 10.1161/CIRCULATIONAHA.118.034309

[75] Wiviott SD, Giugliano RP, Morrow DA, et al. Effect of evolocumab on type and size of subsequent myocardial infarction: a prespecified analysis of the FOURIER randomized clinical trial. JAMA Cardiol. 2020;5(7):787–93. 10.1001/jamacardio.2020.0764. 32347885 10.1001/jamacardio.2020.0764 PMC7191470

[76] Giugliano RP, Pedersen TR, Saver JL, FOURIER Investigators, et al. Stroke prevention with the PCSK9 (proprotein convertase subtilisin-kexin type 9) inhibitor evolocumab added to statin in high-risk patients with stable atherosclerosis. Stroke. 2020;51(5):1546–54. 10.1161/STROKEAHA.119.027759. 32312223 10.1161/STROKEAHA.119.027759

[77] Chiang CE, Schwartz GG, Elbez Y, ODYSSEY OUTCOMES Investigators, et al. Alirocumab and cardiovascular outcomes in patients with previous myocardial infarction: prespecified subanalysis from ODYSSEY OUTCOMES. Can J Cardiol. 2022;38(10):1542–9. 10.1016/j.cjca.2022.05.021. 35644332 10.1016/j.cjca.2022.05.021

[78] Ostadal P, Steg PG, Poulouin Y, ODYSSEY OUTCOMES Investigators, et al. Metabolic risk factors and effect of alirocumab on cardiovascular events after acute coronary syndrome: a post-hoc analysis of the ODYSSEY OUTCOMES randomised controlled trial. Lancet Diabetes Endocrinol. 2022;10(5):330–40. 10.1016/S2213-8587(22)00043-2. 35378068 10.1016/S2213-8587(22)00043-2

[79] Deedwania P, Murphy SA, Scheen A, et al. Efficacy and safety of PCSK9 inhibition with evolocumab in reducing cardiovascular events in patients with metabolic syndrome receiving statin therapy: secondary analysis from the FOURIER randomized clinical trial. JAMA Cardiol. 2021;6(2):139–47. 10.1001/jamacardio.2020.3151. 32785614 10.1001/jamacardio.2020.3151 PMC9426725

[80] Imbalzano E, Ilardi F, Orlando L, Pintaudi B, Savarese G, Rosano G. The efficacy of PCSK9 inhibitors on major cardiovascular events and lipid profile in patients with diabetes: a systematic review and meta-analysis of randomized controlled trials. Eur Heart J Cardiovasc Pharmacother. 2023;9(4):318–27. 10.1093/ehjcvp/pvad019. 36972610 10.1093/ehjcvp/pvad019

[81] Sabatine MS, Leiter LA, Wiviott SD, et al. Cardiovascular safety and efficacy of the PCSK9 inhibitor evolocumab in patients with and without diabetes and the effect of evolocumab on glycaemia and risk of new-onset diabetes: a prespecified analysis of the FOURIER randomised controlled trial. Lancet Diabetes Endocrinol. 2017;5(12):941–50. 10.1016/S2213-8587(17)30313-3. 28927706 10.1016/S2213-8587(17)30313-3

[82] Carugo S, Sirtori CR, Corsini A, Tokgozoglu L, Ruscica M. PCSK9 inhibition and risk of diabetes: should we worry? Curr Atheroscler Rep. 2022;24(12):995–1004. 10.1007/s11883-022-01074-y. 36383291 10.1007/s11883-022-01074-y PMC9750910

[83] Goldman A, Raschi E, Cukierman-Yaffe T, et al. Hyperglycaemic disorders associated with PCSK9 inhibitors: a real-world, pharmacovigilance study. Eur J Prev Cardiol. 2022;29(9):1334–42. 10.1093/eurjpc/zwab209. 34897409 10.1093/eurjpc/zwab209

[84] Khan SU, Rahman H, Okunrintemi V, et al. Association of lowering low-density lipoprotein cholesterol with contemporary lipid-lowering therapies and risk of diabetes mellitus: a systematic review and meta-analysis. J Am Heart Assoc. 2019;8(7):e011581. 10.1161/JAHA.118.011581. 30898075 10.1161/JAHA.118.011581 PMC6509736

[85] Guedeney P, Giustino G, Sorrentino S, et al. Efficacy and safety of alirocumab and evolocumab: a systematic review and meta-analysis of randomized controlled trials. Eur Heart J. 2022;43(7):e17–25. 10.1093/eurheartj/ehz430. 31270529 10.1093/eurheartj/ehz430

[86] Marston NA, Gurmu Y, Melloni GEM, et al. The effect of PCSK9 (proprotein convertase subtilisin/kexin type 9) inhibition on the risk of venous thromboembolism. Circulation. 2020;141(20):1600–7. 10.1161/CIRCULATIONAHA.120.046397. 32223429 10.1161/CIRCULATIONAHA.120.046397 PMC7469753

[87] Bergmark BA, O’Donoghue ML, Murphy SA, et al. An exploratory analysis of proprotein convertase subtilisin/kexin type 9 inhibition and aortic stenosis in the FOURIER trial. JAMA Cardiol. 2020;5(6):709–13. 10.1001/jamacardio.2020.0728. 32347887 10.1001/jamacardio.2020.0728 PMC7301224

[88] Bonaca MP, Nault P, Giugliano RP, et al. Low-density lipoprotein cholesterol lowering with evolocumab and outcomes in patients with peripheral artery disease: insights from the FOURIER trial (further cardiovascular outcomes research with pcsk9 inhibition in subjects with elevated risk). Circulation. 2018;137(4):338–50. 10.1161/CIRCULATIONAHA.117.032235. 29133605 10.1161/CIRCULATIONAHA.117.032235

