# Supplementary material for: PCSK9 and Lipid Metabolism: Genetic Variants, Current Therapies, and Cardiovascular Outcomes
Source: Cardiovasc Drugs Ther. 2024 Jun 22;39(6):1439–51. doi: 10.1007/s10557-024-07599-5 (PMC12717104; doi:10.1007/s10557-024-07599-5)
Supplement: Supplementary file 1 — Supplementary file1 (DOCX 33 kb) [file 10557_2024_7599_MOESM1_ESM.docx]

**Cardiovascular Drugs and Therapy**

**PCSK9 and lipid metabolism: genetic variants, current therapies, and cardiovascular outcomes**

Daniela Grejtakova^1^, Iveta Boronova^1^, Jarmila Bernasovska^1^, Stefano Bellosta^2^

^1^ Laboratory of Molecular Genetics, Department of Biology, Faculty of Humanities and Natural Sciences, University of Presov, 17 November 1, 08001, Slovakia

^2^ Department of Pharmacological and Biomolecular Sciences "Rodolfo Paoletti," Università degli Studi di Milano, Via Balzaretti 9, 20133, Milan, Italy

**Corresponding author**

Daniela Grejtakova, PhD.

Department of Biology, Faculty of Humanities and Natural Sciences, University of Presov, Slovakia

Email: [daniela.grejtakova@gmail.com](mailto:daniela.grejtakova@gmail.com); daniela.grejtakova@unipo.sk

ORCID: 0000-0003-4688-2662

**List of most clinically significant *PCSK9* genetic variations and their characteristics**

| **Reference** | **Exon** | **Nucleotide/protein position** | **Functional effect** | **MAF** | **Population** | **LDL-C** | **CHD risk** | **VLDL** | **ApoA** | **ApoB total** | **ApoB48** | **Lp(a)** | **HDL** | **Clinical features** | **Fasting TG** | **Circulating PCSK9** |  |
| --- | --- | --- | --- | --- | --- | --- | --- | --- | --- | --- | --- | --- | --- | --- | --- | --- | --- |
|  |  |  |  |  |  |  |  |  |  |  |  |  |  |  |  |  |  |
| [4, 30] | 2 | c.380G>C S127R | GOF | < 0.01 | Caucasian; African | 279 ±59 | ↑ | ↑three-fold |  | apoB100 ↑three-fold |  |  |  | tendon xanthomas; CHD; early MI stroke |  | ↑ |  |
|  |  |  |  |  |  |  |  |  |  |  |  |  |  |  |  |  |  |
| [35] | 7 | c.1120G>T D374Y | GOF | < 0.01 | Anglo-Saxon | 264±65-350±97; ↑10–25-fold higher affinity of PCSK9 for the LDLR | ↑ |  |  |  |  |  |  | Achilles tendon xanthomas; premature CHD; more stringent treatment |  | ↓ |  |
|  |  |  |  |  |  |  |  |  |  |  |  |  |  |  |  |  |  |
|  |  |  |  |  |  |  |  |  |  |  |  |  |  |  |  |  |  |
| [31, 32, 33, 89] | 12 | c.2009G>A E670G | GOF | 0.36 | Canadian-Caucasian; Japenese; Tunisian |  | ↑ |  | ↑ | ↓ |  |  | ↑ | stenosis ≥50 % in two or three major coronary arteries | 1.12±0.41 mmol/L; ↓27.7% |  |  |
| [23, 91] | 1 | c.94G>A E32K | GOF |  | Japenese | 2.10-fold higher in homozygotes when mutation in LDLR presented/ ↑homoz. 339mg/dL vs.heteroz. 222-248mg/dL |  |  |  |  |  |  | 49.9±12.1 mg/dl |  | ↑115.5 ±59.7 mg/dl | ↑30% |  |
|  |  |  |  |  |  |  |  |  |  |  |  |  |  |  |  |  |  |
|  |  |  |  |  |  |  |  |  |  |  |  |  |  |  |  |  |  |
| [34] | 10 | c.1547G>T G516V | GOF |  | South Africa | 236±73mg/dL ↑39 mg/dL only for the G516V mutation vs. noncarriers ↑tendon xanthoma | ↑ |  |  |  |  | 54 (41–83) mg/dL | 54±18 mg/dL |  | 142±106 mg/dL | 218±45 ng/mL |  |
|  |  |  |  |  |  |  |  |  |  |  |  |  |  |  |  |  |  |
|  |  |  |  |  |  |  |  |  |  |  |  |  |  |  |  |  |  |
|  |  |  |  |  |  |  |  |  |  |  |  |  |  |  |  |  |  |
|  |  |  |  |  |  |  |  |  |  |  |  |  |  |  |  |  |  |
|  |  |  |  |  |  |  |  |  |  |  |  |  |  |  |  |  |  |
| [1] | 3 | c.426C>G Y142X | LOF | 0.8; < 1 | African Americans; European American | 103±3.9 ↓40% | ↓88% |  |  |  |  |  |  |  |  |  |  |
|  |  |  |  |  |  |  |  |  |  |  |  |  |  |  |  |  |  |
| [1] | 12 | c.2037C>A C679X | LOF | 1.8; < 1 | African Americans; European American | 100±4.5 ↓40% | ↓88% |  |  |  |  |  |  |  |  |  |  |
|  |  |  |  |  |  |  |  |  |  |  |  |  |  |  |  |  |  |
| [17] | 1 | c.137G>T R46L | LOF | 1.8-3.2 | Caucasian | 1.93-3.05 mmol/L ↓15%/↓10% in FH subjects 2.94 mmol/L after OFL | ↓47% | fasting 0.35 mmol/L | 1.19± 0.17g/L | 0.12-0.57g/L | 6.07 μg/mL | 8-9 mg/dl | 70mg/dl; 1.50± 0.29 mmol/L | ↓12% non-HDL in apoE3/E2 ↑2-fold increase in insulin | 1.25-3.62mmol/L; 115 mg/dl | 70.65-241±67 ng/ mL/ after OFL 183±42 ng/ mL 15% -22% |  |
|  |  |  |  |  |  |  |  |  |  |  |  |  |  |  |  |  |  |
|  |  |  |  |  |  |  |  |  |  |  |  |  |  |  |  |  |  |
| [39, 93] | 1 | L15 -L21ins InsLEU | LOF | 0.23 | French Canadian/ Caucasian | 2.27± 0.64 mmol/L; ↓23% | ↓ |  | 1.19± 0.48g/L | 0.65±0.18 g/L |  |  | 1.30± 0.26 mmol/L | increased occurrence of prediabetes and diabetes status | 0.85mmol/L | 82.29± 24.60 ng/mL |  |
|  |  |  |  |  |  |  |  |  |  |  |  |  |  |  |  |  |  |
| [17, 33] | 9 | c.1420G>A I474V | LOF | 0.3 | African; Caucasian | 3.05 mmol/L 2.94 mmol/L after OFL | ↓ |  |  |  | 6.07 μg/mL |  |  |  | 1.67 ± 0.81 mmol/L | 241±67 ng/mL/after OFL 183±42 ng/ mL |  |
|  |  |  |  |  |  |  |  |  |  |  |  |  |  |  |  |  |  |
| [17] | 1 | c.158C>T A53V | LOF | 0.23 | Caucasian, Asian | 3.05 mmol/L 2.94 mmol/L after OFL |  |  |  |  | 6.07 μg/mL |  |  |  |  | 241±67 ng/mL/after OFL 183±42 ng/ mL |  |
|  |  |  |  |  |  |  |  |  |  |  |  |  |  |  |  |  |  |
| [90] | 3 | c. 456G> C Q152H | LOF |  | French Canadian/ Caucasian | 48%↓/ 2.11mM | ↓ |  |  | 0.84g/L |  |  |  | delayed dietary TG clearance, insulin resistance in obese subjects |  | 126ng/ml ↓79% |  |
|  |  |  |  |  |  |  |  |  |  |  |  |  |  |  |  |  |  |
| [92] | 1 | c.202delG Ala68fsLeu82X | LOF | 0.01-0.03 | Caucasian | 0.62-0.96 mmol/L | ↓ |  |  | 28-40 mg/dL |  |  | 1.34- 0.75 mmol/L |  | 0.42-1.83 mmol/L |  |  |

*Abbreviations:* LDL-C - Low density lipoprotein cholesterol; VLDL-C -very low-density lipoprotein cholesterol; ApoA- apolipoprotein A; ApoB - apolipoprotein B; TG-Triglycerides; Lp(a) - Lipoprotein a; HDL-C - high density lipoprotein cholesterol; non- HDL - non-high density lipoprotein cholesterol (including the cholesterol content present in all atherogenic lipoproteins); OFL-Oral Fat Load
