# Supplementary material for: PCSK9 and Lipid Metabolism: Genetic Variants, Current Therapies, and Cardiovascular Outcomes
Source: Cardiovasc Drugs Ther. 2024 Jun 22;39(6):1439–51. doi: 10.1007/s10557-024-07599-5 (PMC12717104; doi:10.1007/s10557-024-07599-5)
Supplement: Supplementary file 2 — Supplementary file2 (PPTX 43 kb) [file 10557_2024_7599_MOESM2_ESM.pptx]

## Slide 1
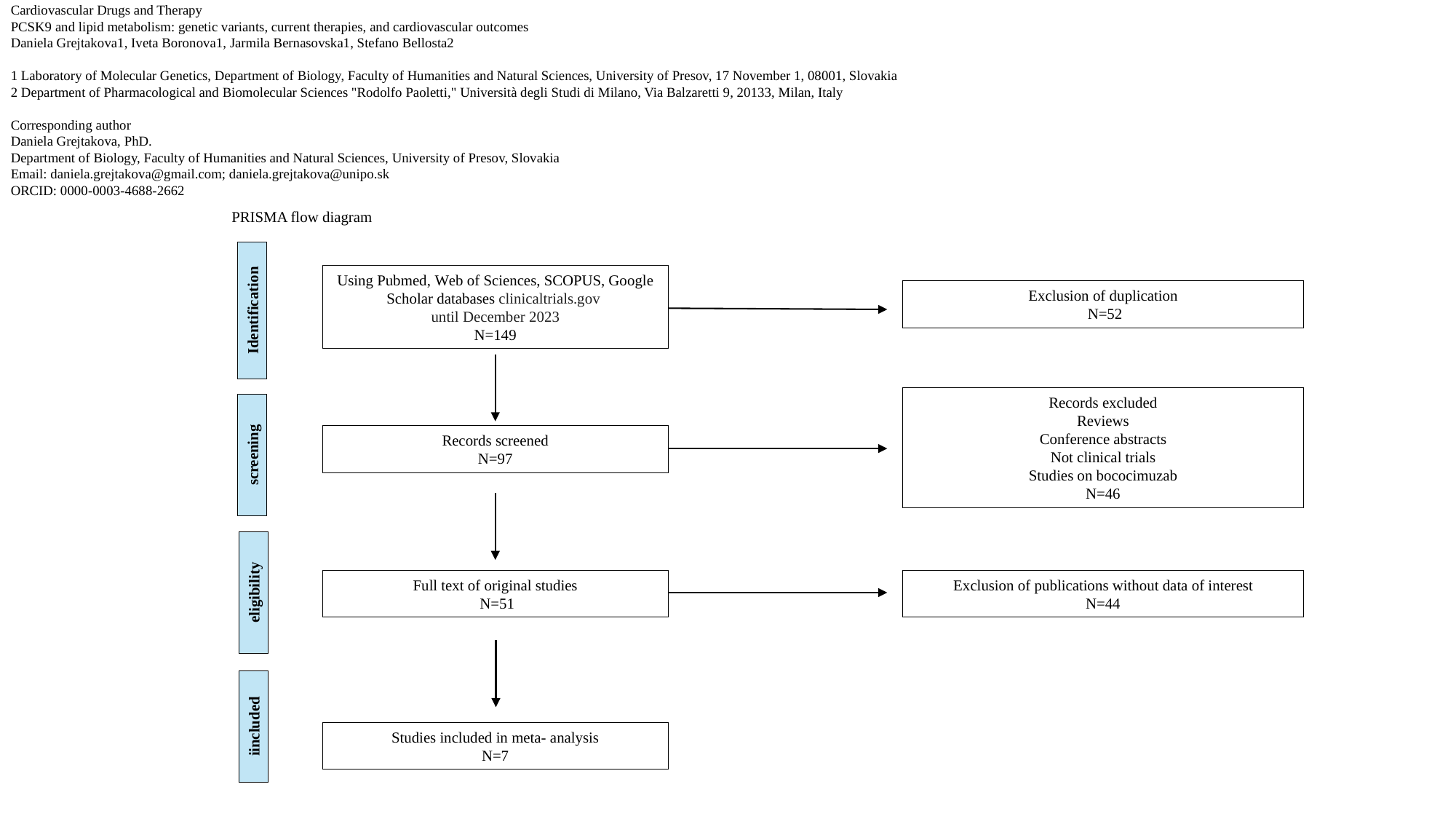

Cardiovascular Drugs and Therapy
PCSK9 and lipid metabolism: genetic variants, current therapies, and cardiovascular outcomes
Daniela Grejtakova1, Iveta Boronova1, Jarmila Bernasovska1, Stefano Bellosta2
1 Laboratory of Molecular Genetics, Department of Biology, Faculty of Humanities and Natural Sciences, University of Presov, 17 November 1, 08001, Slovakia
2 Department of Pharmacological and Biomolecular Sciences "Rodolfo Paoletti," Università degli Studi di Milano, Via Balzaretti 9, 20133, Milan, Italy
Corresponding author
Daniela Grejtakova, PhD.
Department of Biology, Faculty of Humanities and Natural Sciences, University of Presov, Slovakia
Email: daniela.grejtakova@gmail.com; daniela.grejtakova@unipo.sk
ORCID: 0000-0003-4688-2662
PRISMA flow diagram
Using Pubmed, Web of Sciences, SCOPUS, Google Scholar databases clinicaltrials.gov
until December 2023
N=149
Exclusion of duplication
 N=52
Identification
Records excluded
Reviews
Conference abstracts
Not clinical trials
Studies on bococimuzab
N=46
Records screened
N=97
screening
Full text of original studies
 N=51
Exclusion of publications without data of interest
N=44
eligibility
iincluded
Studies included in meta- analysis
N=7
